# Recent Advances in the Determination of Major and Trace Elements in Plants Using Inductively Coupled Plasma Optical Emission Spectrometry

**DOI:** 10.3390/molecules29133169

**Published:** 2024-07-03

**Authors:** Marin Senila

**Affiliations:** INCDO-INOE 2000, Research Institute for Analytical Instrumentation, 67 Donath Street, 400293 Cluj-Napoca, Romania; marin.senila@icia.ro

**Keywords:** metal determination, heavy metal, ICP-OES, vegetable, acid digestion, microwave digestion, combustion, green analytical method, extraction technique

## Abstract

Interest in measuring major and trace elements in plants has increased in recent years because of growing concerns about the elements’ contribution to daily intakes or the health risks posed by ingesting vegetables contaminated by potentially toxic elements. The recent advances in using inductively coupled plasma atomic emission spectrometry (ICP-OES) to measure major and trace elements in plant samples are reviewed in the present work. The sample preparation before instrumental determination and the main advantages and limitations of ICP-OES are described. New trends in element extraction in liquid solutions using fewer toxic solvents and microextractions are observed in recently published literature. Even though ICP-OES is a well-established and routine technique, recent innovations to increase its performance have been found. Validated methods are needed to ensure the obtaining of reliable results. Much research has focused on assessing principal figures of merit, such as limits of detection, quantification, selectivity, working ranges, precision in terms of repeatability and reproducibility, and accuracy through spiked samples or certified reference materials analysis. According to the published literature, the ICP-OES technique, 50 years after the release of the first commercially available equipment, remains a powerful and highly recommended tool for element determination on a wide range of concentrations.

## 1. Introduction

The determination of mineral elements is a critical aspect of chemical analysis in most types of samples. Even though about three-quarters of the elements in the periodic table are metals, only several are known as being essential for living organisms due to their biochemical role in the human body. Elements like Ca, K, P, Na, and Mg are major essential elements, whereas elements such as Fe, Zn, Mn, Cu, and Se are trace essential elements [1,2,3,4]. The deficiencies of these elements may cause malfunctioning of organisms, while their concentration above certain thresholds may negatively affect the organism’s health [5,6]. Other elements (Cd, Pb, Hg, As, and Sr) with no known biological function represent a health risk even at low concentrations [7,8,9].

Amongst the most widespread methods for determining major and trace elements, spectrometric methods based on inductively coupled plasma (ICP), like inductively coupled plasma–optical emission spectroscopy (ICP-OES) and inductively coupled plasma-mass spectrometry (ICP-MS), are known for their robustness, low detection limits, good accuracies, large linear ranges of concentrations, low detection limits, and multielement determination capability [10,11,12,13,14]. The robustness of the ICP-OES technique is also demonstrated by the fact that 50 years after the first commercial instrument appeared [10], it remains one of the most widely used techniques for determining major and trace elements in various types of liquid and solid samples.

In ICP-OES, plasma is used to emit photons with characteristic wavelengths for each analyzed element, ensuring thus the element’s identification, while the intensity of emitted radiation is proportionally linked to the concentration of the analyte. The samples typically need to be introduced into the plasma in their liquid form by nebulization. Therefore, the analysis of vegetables implies their digestion to bring analytes from their solid matrix into a liquid aqueous solution. In the case of vegetable analysis by ICP-OES, the sample preparation usually includes several steps, such as washing/cleaning, drying, crushing, sieving, digestion using a mixture of acids, filtration, and then measurement by ICP-OES [15]. Thus, plant sample preparation before ICP-OES analysis has been extensively studied.

Given the general tendency in analytical chemistry to achieve greener methodologies, determining chemical elements by inductively coupled plasma-based techniques should become as environmentally friendly as possible. Anastas [16] first drew attention to the necessity of adapting analytical methodologies to the requirements of green chemistry. Nowak et al. [17] introduced the concept of white analytical chemistry, while Gałuszka et al. [18] formulated the 12 main principles of green analytical chemistry to protect the environment and analysts while performing analytical procedures. The primary strategy of this concept comprises reducing the stages of analytical procedures, performing on-site analysis using portable instruments, replacing or eliminating toxic reagents in sample preparation or sampling procedures, minimizing the use of energy, performing multi-parameter analysis, and increasing the safety of analysts [18,19]. Thus, one of the aims of this review was also to assess the possible integration of metals analysis in vegetables by ICP-OES in the principles of green analytical chemistry, both from the instrumentation and from sample preparation points of view.

This review aims to present comprehensive information on the new trends and findings from ICP-OES application in plant analysis. It emphasizes the necessity and importance of ensuring appropriate quality control in plant analysis and characterization. The literature published in the last ten years was mostly considered.

## 2. Plants Samples Preparation for ICP-OES Analysis

**Figure 1:**
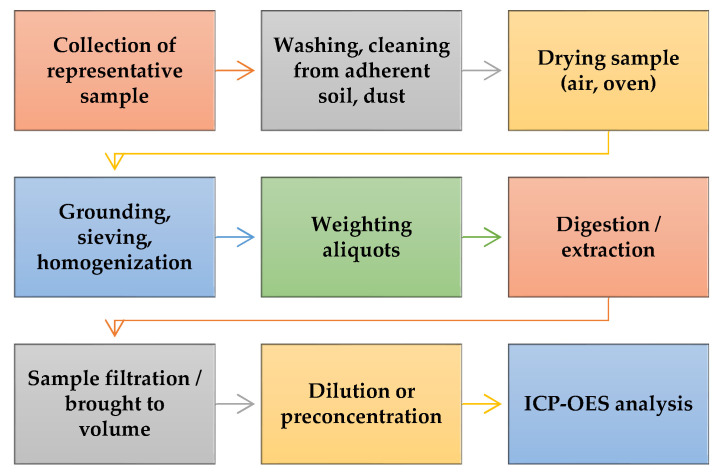
Summary of steps for plant sample preparation process for ICP-OES determination.

Figure 1 presents a schematic representation of the analytical steps required for determining major and trace elements in plants by ICP-OES.

Sample preparation is a critical issue for obtaining representative and consistent results. Firstly, the samples should be representative of the intended study. Depending on the scope, the edible part should be collected [20,21,22]. Next, the samples must be cleaned and washed with tap water and then distilled/deionized water to eliminate dust and soil-adhering particles [23,24]. To obtain the dry mass of plant samples, these can be dried using different approaches, like air-drying [23,25] for days to weeks, drying in an oven at constant temperature for hours to days until constant weight [26,27,28,29], or freeze-drying [22]. When dried in the oven, generally temperatures of 50–80 °C are used to ensure faster water evaporation and low enough temperatures to avoid possible loss of the analytes. The dried samples are powdered with grinders, blenders, and agate/porcelain mortars and pestles [30,31]. The plant sample powder obtained by grinding is often directly digested, while other authors have sieved the powders before digestion [32,33].

**Figure 2:**
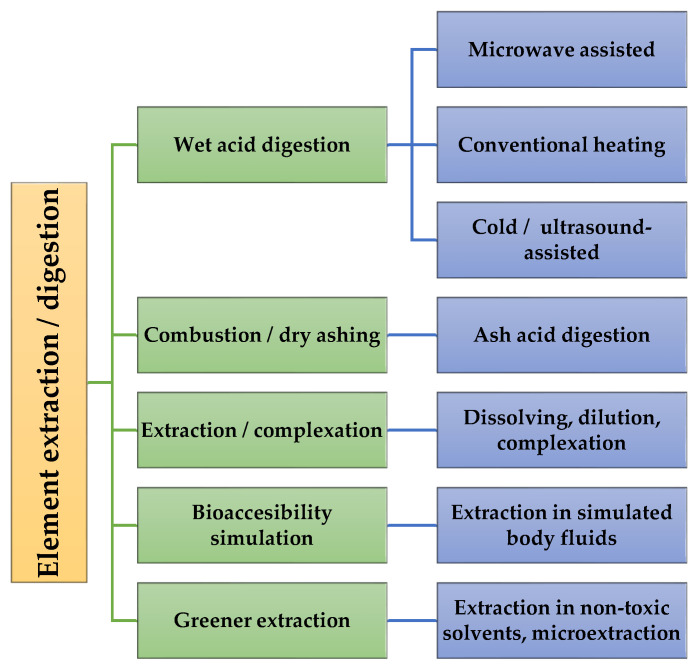
Classification of main element extraction procedures from plant powders for ICP-OES analysis.

Different approaches have been developed and presented in the literature to extract analytes from solid plant samples to liquid solutions. These are typically focused on five methodologies: (1) wet acid digestion; (2) combustion, followed by ash acid digestion; (3) extraction to liquids with complexing chemicals; (4) extraction in simulated body fluids for bioaccessibility studies; and (5) extraction using non-toxic solvents or use of microextraction, on trend with greener sample preparation methods. These approaches are summarized in Figure 2.

The commonly used methodologies for element extraction from solid plants are based on matrix digestion. This can be carried out directly on powdered plant samples using oxidizing acids to destroy organic matter and minimize spectral interferences. Nitric acid is frequently used due to its oxidizing role and because some elements form soluble nitrates. Also, mixtures of HNO_3_ with H_2_O_2_, HCl, HClO_4_, HF, or H_2_SO_4_ are used for sample mineralization [24,34,35]. Depending on the matrix and the analyte of interest, different optimizations of the composition of the mixtures used for digestion and the conditions for wet digestion were carried out. Good digestion efficiency is obtained if the organic components of the samples are removed. In this sense, sample combustion prior to acid extraction can be employed, even though this involves supplementary steps and is a possible source of contamination.

### 2.1. Wet Acid Digestion of Plant Samples for Metals Determination

Table 1 provides examples of wet acid digestion procedures for element extraction from plant samples before their instrumental determination from selected literature published from 2014 to 2024.

Even though acid–wet digestion can be performed on a hot plate or in closed microwave systems, microwave-assisted digestion was chosen in most studies. The use of microwave conditions with closed vessels has several advantages, since the time for digestion is shorter, while the contamination or the loss of analytes is minimized. Moreover, the high pressure and temperature obtained in closed vessels contribute to the degradation of organic matter; thus, the combustion step is not necessary. On the other hand, a lower mass of sample can be digested in closed vessels, typically in the range of 0.1–0.5 g, because the high amount of organic matter increases the pressure in the closed vessels. Conversely, heating on a hotplate in an open vessel has the advantage of digesting higher amounts of sample (reported up to 5–10 g) [39], which represents an advantage in analyzing a more representative sample and in obtaining lower limits of quantification, which is an essential aspect in the measurement of trace elements by ICP-OES.

Even though, in some cases, only HNO_3_ or mixtures of mineral acids were used for digestion [7,32,41,52,67], in most of the published papers, H_2_O_2_ was used as an oxidant agent for the wet digestion of organic matter [13,68,69,70,71,72,73]. Some authors [37] used only H_2_O_2_ to digest the samples, but in particular conditions: single-reaction chamber microwave system that allows temperature up to 300 °C and pressure up to 199 bars. However, digestion based on only H_2_O_2_ is well in agreement with green analytical chemistry recommendations due to the low acidity of resulted solutions and residues [73]. Thus, it is highly recommended for future developments.

It is important to note that in the majority of studies, there is no clear definition of metrics for evaluating the greenness of analytical methodologies. In many cases, the developed analytical methods are considered green by the authors without checking this [74]. To ensure appropriate assessment, several tools have been developed to confirm if a method adheres to green analytical chemistry principles: the National Environmental Methods Index (NEMI) [75], Green Analytical Procedure Index (GAPI) [76], Complementary Green Analytical Procedure Index (ComplexGAPI) [77], Analytical Eco-Scale (AES) [78], Analytical Method Greenness Score (AMGS) [79], Analytical Greenness Metric (AGREE) [80], and the Analytical Greenness Metric for Sample Preparation (AGREEprep) [81]. On the topic of metals and metalloids analysis by ICP-OES following sample digestion, the existing literature on greenness evaluation procedures is scarce. These evaluations are predominantly applied to chromatographic methods, which typically involve the use of higher quantities of chemicals [82]. However, in several papers, the authors used the abovementioned tools to assess the green character of developed methods. For instance, Pereia Junior et al. [83] developed a sample preparation method for the determination of As, Ca, Cd, Cu, Cr, K, Fe, P, Pb, Mg, Mn, Na, Sr, and Zn in medicinal herbs by digestion in a closed digester block prior to ICP-OES measurement. The optimized parameters for digesting 0.10 g of a medicinal herb sample were as follows: a heating period of 120 min at 180 °C was employed, utilizing a mixture comprising 1.38 mL of 65% HNO_3_, 1.00 mL of 30% H_2_O_2_, and 2.62 mL of deionized water. The AGREE metric yielded a score of 0.63, thereby establishing the method’s environmental friendliness [83]. In a study by Ncube et al. [84], a microwave-assisted digestion method was developed for the determination of arsenic, cadmium, chromium, lead, and tin in pet food samples. Hydrogen peroxide was used as a digestion reagent, and subsequent metal determination was conducted using inductively coupled plasma optical emission spectrometry (ICP-OES). The AGREEprep metric instrument was employed by the authors to evaluate the method’s green degree, resulting in a score of 0.76, which confirmed its green nature [84].

### 2.2. Combustion and Acid Digestion

Plant samples contain high amounts of organic substances, so their incineration may be very suitable for sample digestion. Practically, in this way, the organic matrix is eliminated in the form of CO_2_ and H_2_O, while the remaining residue after burning represents inorganic substances that diluted mineral acids can dissolve. Table 2 shows several selected examples of combustion followed by dissolving the resulting residue for element measurement in plant samples.

In general, the methods based on combustion involve relatively simple equipment. The amount of the analyzed sample can be higher than in direct microwave digestion because the decomposition of organic matter is made separately, generally in open vessels, and thus does not produce high pressure. The mass of the resulting ash is much lower than that of the initial sample and can be dissolved with diluted mineral acids [55,89,90,91]. However, this process is longer than direct acid digestion, and the risk of contamination or analyte loss may appear due to the multiple steps involved. Thus, the entire procedure should be carefully conducted.

### 2.3. Dissolving, Complexing, and Green Extraction Methods

Even though the metals were analyzed after acid digestion in most reported studies, several papers reported the extraction of metals with different other types of reagents, or in mixtures of diluted acids. For example, Butorova et al. [25] measured the metals concentration in the ethanol/water extracts.

Deep eutectic solvents (DESs) are newly reported as environmentally friendly solvents for metal extraction from samples with organic matrices, including from plant samples. DESs involve a system formed from a hydrogen bond donor (HBD) and an acceptor (HBA) [92,93]. This system decreases the melting point so that the extraction can be performed even at room temperature. The typical HBA is choline chloride, which is a natural compound. Many substances, such as tartaric, citric, benzoic, oxalic, acetic, malonic, malic, formic, maleic, succinic, adipic, boric, lactic, ascorbic, gallic, and mandelic acids; 1,4-butanediol; glycerol; sorbitol; ethylene glycol; triethylene glycol; benzamide; urea; thiourea; fructose; glucose; sucrose; and maltose have been tested as HDB [92,94]. Table 3 displays some examples of metals extraction from plant samples by extraction with solvents, including DES as green solvents.

The number of published papers on this topic is relatively limited, while the tools for assessing the greenness of analytical methods have rarely been employed. Abellan-Martín and co-workers [101] developed a methodology for the measurement of As, Cd, Hg, and Pb in drugs by ICP OES, based on chemical vapor generation subsequent to dispersive liquid–liquid microextraction using a natural deep eutectic solvent as the extractant. An 50-fold improvement of LOQs was reported. The developed method was demonstrated to have an excellent green character using the AGREEprep metrics, as evidenced by the AGREEprep score of 0.40 [101]. Sihlahla et al. [102] used alcohol-based deep eutectic solvents (DES) for sample digestion and determination of Se by ICP-OES. DES were prepared from choline chloride (ChCl) as a HBA and phenol as a HBD, in different molar ratios. A 0.1 g sample was mixed with 4 mL of the DES and shaken for 3 min using a vortex. The sample was digested for 25 min at 125 °C. Following cooling to room temperature, 4 mL of 3 M NHO_3_ was added. The greenness of the method was evaluated using three metrics tools: NEMI, AES, and AGREE, and it was demonstrated that the developed protocol is an excellent green method [102]. Given the paucity of existing literature on this subject, further research is required to develop more environmentally friendly techniques for the determination of metals by ICP-OES, as well as to assess their sustainability using the existing assessment tools.

### 2.4. Extraction for Bioaccesibility Studies on Plant Samples

The total concentration of metals in vegetal foodstuffs is not totally transferred and absorbed by the human body. Thus, recent studies on metal content in edible plants focused on assessing the fraction of the metal released into the food matrix in similar conditions to those from the gastrointestinal tract that can be transferred to the body. This portion of elements is referred to as bioaccessible concentration [103,104]. Table 4 presents several examples of digestion methods used in bioaccessibility studies.

The studies dealing with the bioaccessibility of metals from different plants used fresh or dried samples, from which metals are extracted in simulated body fluids (SBF) having similar pH and enzymes (pepsin, pancreatin, amylase) with those from gastrointestinal tract, and being kept for a similar time of contact (saliva, pH @ 6.8, 5 min; gastric juice pH = 2–3, 1 h; duodenal juice, pH = 6.5–7.0, 3 h) [41]. This method of analyzing bioaccessible fractions of trace elements is a good surrogate of bioavailable concentration and has received acceptance [105].

## 3. Advantages, Limitations and Advances in Plasma Viewing, Sample Introduction Systems and Miniaturization of Optical Emission Spectrometry Instrumentation

The main advantage of ICP-OES is that it is capable of multielement determination over a wide range of element concentrations, making it a very productive technique compared with atomic absorption-based methods. As the main drawbacks, the limits of detection (LODs) and limits of quantification (LOQs), which are higher than in ICP-MS or GFAAS, make this sometimes not suitable for direct analysis of toxic elements in plants or vegetables used as foodstuffs due to their very low maximum admitted levels. For this reason, efforts have been made in recent years to improve the mentioned parameters by new approaches in producing plasma, sample introduction systems, plasma viewing, or detection systems [10,106,107].

Inductively coupled plasma (ICP) is generated in an inert gas (typically argon) in a torch having three concentric tubes made of quartz or ceramic, with the aid of a radiofrequency (RF) generator and an induction coil [108]. Legally, authorized frequencies for plasma generators are 27.12 MHz and 40.68 MHz, but the frequency of 40.68 MHz is increasingly used in modern equipment because it ensures higher plasma stability and establishes a higher central channel into the ICP, helping in the more accessible introduction of the sample, conducting to increased performance [109].

Concerning plasma viewing, there are two possibilities for observing the light emitted by the plasma: radial and axial view. Both viewing modes have advantages and disadvantages. In radial mode, the analytical signals are lower, which can lead to higher detection limits. In the case of elements found at trace concentrations, this represents a clear disadvantage. However, for major elements or elements with a high sensitivity, this is an advantage, because no dilution of sample is required. Moreover, the background signal is lower in this case; thus, the matrix effect is decreased [109]. Axially viewed plasma has the advantage of collecting all the element emissions over the whole length of the plasma, and thus, the emission path length is enhanced compared to radial view [110]. This has an effect on the increased sensitivity for trace elements, but this comes with the disadvantages of increased background signal and with the signal saturation for analytes in high concentrations or with high sensitivity (e.g., sodium, potassium, lithium, strontium, etc.). For these reasons, one of the advances in ICP-OES instruments was dual viewing (axial and radial). In this approach, the viewing mode can be selected for each specific element, taking advantage of the plasma viewing mode in multielement analysis.

Another advance in ICP-OES systems was made in sample introduction systems. Nebulization efficiency was improved by the development of ultrasonic nebulizers, which generate higher aerosol amounts up to 10-fold. In an ultrasonic nebulizer, the sample is injected into a piezoelectric transducer, which destroys the sample into a homogeneous fine aerosol, decreasing the limits of detections compared to a pneumatic nebulizer [111]. Chemical vapor generation was another approach developed to improve the analytical performances of ICP-OES. In this technique, the analyte is extracted as a gas from the matrix, and it is selectively and more efficiently introduced into the equipment, obtaining excellent improvements in LODs [112,113,114,115].

The miniaturization of ICP-OES equipment is a growing trend in research aimed at making this analytical technique more economically sustainable and practical for on-site applications. The critical aspects of the advances in the miniaturization of ICP-OES instruments are the miniaturized components [116]. Microplasma technology involves the use of microtorches with microplasmas that run at low power consumption and small gas flow rates [117,118,119]. However, this is still at the research level, and future developments are needed for producing commercial equipment.

## 4. Method Validation and Performance Parameters for ICP-OES Used in Plant Sample Analysis

Because digested plant samples comprise complex matrices, ICP-OES measurements need studies on the method’s performance in the validation process to obtain reliable results. In these types of samples, both spectral and non-spectral interference may occur. Other spectral wavelengths can be selected to solve the problem of spectral interferences if the sensitivity is not severely affected. Another possibility is using spectral corrections with spectrometer software, which is available for many commercial instruments. The minimization or removal of non-spectral or matrix interferences is usually obtained in three ways: (1) using “matrix matching” calibration standards for instrument calibration, (2) using the standard addition method, or (3) using internal standards added in blanks, calibration solutions and the samples, with the condition that internal standard is absent in these solutions and has a similar behavior in the plasma to the analytes [120]. However, the method development step should carefully study all three methods to obtain good accuracy. Table 5 gives examples of figures of merit reported for element determination in plants using ICP-OES.

The most studied performance parameters in plant analysis by ICP-OES were LODs/LOQs, precision in terms of repeatability and reproducibility, and accuracy in terms of studying recovery using certified reference materials or spiked samples. Generally, satisfactory performances were reported for accuracy and precision, the two parameters used for quality control in ICP-OES, within the acceptability criteria. Although CRMs with the same matrices as the sample matrix were unavailable, accuracy assessment was reported by analyzing CRMs with similar matrices. For example, Giacomino et al. [60] used a tomato leaf CRM for quality control of vegetal oil analysis, and the recoveries found were 75% to 101.5%. Higher recoveries (93.1–102.7%) were reported by González-Suárez et al. [88] for analysis of four CRMs of bovine liver, apple leaves, typical diet, and wheat flour. At the same time, the standard addition method was employed for recovery assessment for lithium. Sakar et al. [3] obtained recoveries of over 90% for analyzing metals in standard reference materials (SRMs) of tomato leaves and rice flour. Giraldo et al. [121] found that for Cd determination, recovery percentages for ICP-OES were similar to those by ICP-MS (over 90%). Also, similar recoveries (96.0–108.3%) were obtained by ICP-OES and ICP-MS techniques for elements analysis in CRMs after the ultrasound-assisted extraction method [122]. In all studies, the reported recoveries for CRM analysis indicated good accuracies compared with legal requirements [123].

The methods were generally validated in terms of selectivity, sensitivity, limits of detection and quantification, accuracy, and precision prior to their use for real sample analysis [124]. The ICP-OES is a versatile technique, mainly due to its multielement capability, with up to 70 elements measured at the same time and wide working ranges [125].

## 5. Conclusions

ICP-OES has been successfully used to analyze major and trace elements in plant samples. Although it has been 50 years since the first ICP-OES equipment was marketed, the technique remains a fascinating area of research. Of course, this technique can be applied to the analysis of many types of samples, but the analysis of plants is a niche analysis that requires special attention because of their organic matrix, as well as the need to determine trace and ultra-high concentrations. Many recently published papers deal with improving the sample preparation step. Because of the tendency in analytical chemistry to achieve greener methodologies, much research was carried out to replace or eliminate toxic reagents in sample preparation procedures. The regularly used procedures for element extraction from plant samples are based on acid digestion. This is aided by heating on a hot plate or often with microwaves. Because removing the organic matrix accomplishes an increased digestion efficiency, a supplementary step of sample combustion can be applied before acid extraction. Deep eutectic solvents are increasingly studied as environmentally friendly solvents for metal extraction prior to ICP-OES analysis. Another area of research extensively studied in the last years is the assessment of the bioaccessibility of different elements, mainly from plants used as food sources.

Regarding instrumental ICP-OES developments, many efforts have been made to lower LODs and LOQs through new plasma production methodologies, new sample introduction systems, and improvements in plasma viewing and detection systems. The miniaturization of ICP-OES instruments is a flourishing trend in research aimed at making this analytical technique more economical.

ICP-OES, a well-established technique in many laboratories, has been the focus of recent research aimed at validating ICP-OES-based methods to enhance their accuracy and precision. This comprehensive review not only brings together the recent applications of ICP-OES in various vegetable samples but also underscores its outstanding advantages. In conclusion, the ICP-OES continues to be a fascinating area of research, particularly in its potential to reduce initial and maintenance costs, and in its role in the development of greener sample preparation methodologies, a prospect that is sure to inspire our professional colleagues and researchers in the field.

## Figures and Tables

**Table 1:** Examples of wet acid digestion procedures for element extraction from plant samples.

Analytes	Type of Samples	Digestion Method	References
Ag, As, Al, Ba, Bi, Be, Cd, Ca, Cr, Co, Cu, K, Mn, Mg, Na, Fe, Pb, Li, Ga, Mo, Ni, Rb, Sr, Se, Tl, Te, V, Zn	Curcuma	A: HNO_3_ (3 mL), B: HNO_3_ (6 mL) + H_2_O_2_ 1 M (2 mL), C: HNO_3_ (1 mL) + H_2_O (4 mL) + H_2_O_2_ 1 M (2 mL), D: HNO_3_ (6 mL) + H_2_O_2_ 1 M (2 mL), E: HNO_3_ (1 mL) + HCl (3 mL)	[7]
As, Cd, Be, Cr, Co, Cu, Mn, Fe, Mo, Ni, Sb, Pb, Sn, Se, V, Tl, Zn	Potatoes	0.65 g sample digested with HNO_3_ 70% and H_2_O_2_ 30%, microwave oven, heating program up to 200 °C, total time 23 min	[20]
B, Cd, Ca, Cu, Cr, Fe, K, Mn, Mg, Na, Pb, Ni, Zn	Ten fruit-type plants belonging to the Solanaceae and Cucurbitaceae families	8 mL HNO_3_ 65% added to 0.2 g of plant, digestion in microwave oven, heating program up to 155 °C, total time 20 min	[21]
Al, Mg, Ca, Na, K, As, Co, Cd, Cu, Cr, Li, Fe, Mo, Mn, Pb, Ni, Se, Sb, Sr, Si, Tl, Ti, V Zn	43 plant species	10 mL HNO_3_ added to 0.1–2.1 g plant, digestion in microwave oven	[22]
Cd	Chinese cabbage	Mixture of HNO_3_-HClO_4_, 9:4 (*v*/*v*) ratio, hotplate at temperature of 150–180 °C, until obtaining clear liquid	[23]
Hg, Pb	Different vegetables and herbs	1 g of dried plant digested with 15 mL mixture of HNO_3_ (70%)—H_2_SO_4_ (65%)—HClO_4_ (70%) = 5:1:1 ratio	[26]
As, Co, Ca, Cu, Cr, Fe, Mn, Mg, Pb, Ni, Se, Zn	*Laportea alatipes*	0.25 g dried plant sample digested with 10 mL HNO_3_ 70%, microwave oven	[27]
Cd, Mn, Al and Mg	*Atriplex portulacoides*, *Ulva lactuca*, *Arthrocnemum indicum*	Cold extraction HNO_3_ (1% and 10%) Heating with mixtures of acids: HCl-HNO_3_, HNO_3_-H_2_SO_4_, HNO_3_-HCl-H_2_SO_4_, HNO_3_-H_2_SO_4_-HClO_4_, HNO_3_-HClO_4_, HNO_3_-HCl-HClO_4_	[29]
Pb, Cd	Different fruit and vegetable produce (~1300 samples)	0.2–0.3 g dried plant sample digested with 2.5 mL HNO_3_ conc., then incubated overnight. After, 2.5 mL of H_2_O_2_ was added, microwave-assisted digestion	[31]
Al, As, B, Cd, Co, Cu, Fe, Pb, Se, Zn	Wheat, cabbage, spinach	0.5 g of dried plant mixed with 70% HNO_3_ and heated up to 70 °C. Then 2 mL of HClO_4_ and 2 mL of HNO_3_ was added and heat up at 135 °C for 25 min. After cool down, 2 mL of HNO_3_ and 5 mL of HCl were added, then diluted at 50 mL with water	[32]
As, Cd, Cr, Cu, Ni, Mn, Fe, Pb, Zn	Sweet potato	0.2 g of dried sample digested with 6 mL HNO_3_ 69% and 2 mL of H_2_O_2_ 30%, microwave oven	[33]
Pb, Cr, Cu, Fe, Zn, and Ni	Coriander, parsley, dill, arugula	0.5 g of sample digested with 8 mL HNO_3_ 69%, and let overnight at room temperature. After, 2 mL of H_2_O_2_ 30% were added, and heated on hot plate at 90–120 °C	[36]
Ca, Cu, Fe, Mg, Mn, K, Na and Zn)	Medical foods	1 g of sample + 6 mL H_2_O_2_ 50% at 250 °C and 160 bar, in a single reaction chamber system	[37]
As, Cd, Co, Cr, Cu, Mn, Mo, Ni, Pb, Sb, and Sn	Tomato, onion, pepper, spinach, carrots, lettuce, marrow squash	0.5 g of sample digested with 6 mL HNO_3_ conc. + 2 mL H_2_O_2_ 30%, in microwave oven, five-steps digestion program	[38]
Cd, Pb, As, Hg	Okra, tomato, pumpkin, potato, cabbage, eggplant, spinach	10 mL HNO_3_ conc. added to 5–10 g sample, on hotplate at temperature of 120 °C for 6 h. About 1 mL H_2_O_2_ was periodically added, until a clear solution was obtained	[39]
Ca, K, Mg, P, Na, B, Fe, Mn, Zn, Al, Sr, Co, Cu, Ni, Se, V, As, Cd, Cr, Pb, Sb, Sc, Y	Kale, rapeseed	0.1 g of dried sample digested with 2.5 mL HNO_3_ (70%) and 1 mL H_2_O_2_ (15%), microwave oven	[40]
Al, Cd, Cu, Co, Cr, Fe, Mg, Mn, Ni, Pb, Zn	Medicinal herbs	0.2 g of dried sample digested with 10 mL of HNO_3_:HClO_4_ (2:5 *v/v*) mixture. Few hours cold digestion, then heated on a hot plate, until colorless solution	[41]
As, Cd, Cr, Cu, Mn, Pb, Zn	Coriander, celery, C. coronarium, spinach, leek	0.5 g of dried sample digested with 5 mL HNO_3_ and 1 mL of H_2_O_2_, kept overnight. Then were heated in oven at 150 °C near dryness. Next, 5 mL of HNO_3_ and 2 mL of H_2_O_2_ were added, then kept at 150 °C for 4 h	[42]
Pb, Cd	Lettuce, apples, carrots, tomatoes	0.5 g of vegetable powder mixed with 20 mL of 2.5 M HNO_3_. The mixture was immersed in ultrasonic bath for 15 min. For comparison, microwave digestion was applied: 0.5 g of sample digested with 10 mL of 4.8 mol L^−1^ HNO_3_	[43]
Ca, Mg, K, Na, S, P, Al, As, Ba, Co, Cd, Cr, Cu, Fe, Hg, Ni, Mn, Pb, V, Se, Zn	Brussels sprout, cabbage, potato, onion, kohlrabi, carrot, beetroot	0.5 g of dried sample digested with a mixture HNO_3_:H_2_O_2_ (7:1), heating at 80 °C in a water bath, for 5 h	[44]
As, Cd, Pb, Cr	Ten species of edible plant samples	0.5 g of dried sample digested with 5 mL HNO_3_ and 3 mL H_2_O_2_, heating at 120 °C on a hot plate to near dryness, then was diluted with water to 25 mL	[45]
As, Cr, Cd, Cu, Co, Pb, Ni	Kale, collard greens, basil, romaine lettuce, carrot, potato, radish, tomato, squash, pepper	0.5 g of dried sample digested with 5 mL HNO_3_, microwave oven, at a temperature of 175 °C	[46]
Cd, Cu, Cr, Mn, Pb, Hg	Indocalamus leaves	0.3 g of dried sample digested in two ways: (1) 5 mL HNO_3_ + 1 mL H_2_O_2_; (2) 5 mL HNO_3_ + 1 mL HF, microwave oven	[47]
Cu, Cd, Ni, Pb	59 medicinal plants	0.3 g of dried sample digested with a mix of acids (HNO_3_, HClO_4_, HCl) and H_2_O_2_, microwave oven	[48]
Al	Rice, corn, wheat, rye, barley, triticale, soy, oats	0.5 g of dried sample digested with 1.5 mL H_2_O_2_ and 7 mL HNO_3_, microwave oven	[49]
Al, As, Cd, Cr, Cu, Ni, Zn, Hg, Pb	*Xanthium strumarium* L., *Ficus exasperata*, *Persicaria attenuata*, *Kanahia laniflora*	0.25 g of dried sample digested with 7 mL HNO_3_ (63%) and 2 mL H_2_O_2_ (30%) microwave oven, four-step digestion procedure. After digestion, the samples were evaporated down to 1 mL on a hot plate, then diluted with water to 50 mL and filtered	[50]
As, Cr, Cd, Pb	16 species of edible vegetables	0.2 g of dried sample digested with mixture of 2 mL 1:1 (*v*/*v*) HNO_3_:H_2_O, heated at 90 °C on a hotplate; 1 mL HNO_3_ was repeatedly added until brown fumes disappeared. Sample was evaporated to 1 mL, then 0.4 mL H_2_O and 0.6 mL 30% H_2_O_2_ were added and heated again until effervescence stopped	[51]
As, Cd, Pb, Ni, Fe, Zn	Traditional medicine samples	0.5 g of dried sample digested three ways: (1) 5 mL HNO_3_ + 2.5 mL HClO_4_; (2) 5 mL HNO_3_ + 2.5 mL HNO_3_; (3) 9 mL mixture HNO_3_:HCl (1:3), heating until total dissolving	[52]
Cu, Cd, Cr, Ni, Fe, Pb, Mn, Zn	*Urtica urens*	2 g of dried sample digested with 25 mL 5% HNO_3_ heating by induction then cooled down. Afterward, 15 mL of 5% HClO_4_ was added and boiled 1 until the solution became colorless	[53]
Cd, Cu, Cr, Ni, Pb	*Atriplex leucoclada*, *Salsola imbricata*, *Typha augustifolia*, *Calotropis procera*, *Phragmites australis*	0.5 g of dried sample digested with HNO_3_ and H_2_O_2_, using a large-capacity HotBlock digestion system by heating until clear solutions were obtained	[54]
Ba, Be, Bi, Ca, Co, Cs, Cu, Mg, Mn, Na, K, P, Pb, Ni, Rb, Sr, Mo, Th, U, Zn, REEs	Botanical samples	0.2 g sample digested in microwave-assisted conditions. 2.5 mL of conc. HNO_3_ was added for predigestion 4 h. Then, 2 mL of 30% H_2_O_2_ was added for digestion in a microwave oven	[55]
14 rare earth elements (REEs)	Chinese cabbage, long bean, towel gourd, scallion, radish, white gourd, eggplant, potato, tomato, carrot, red pepper, pumpkin	0.50 g of dried sample digested with 8 mL of HNO_3_ (65%), microwave oven, cooled down, and then diluted to 10 mL with ultrapure water	[56]
Ca, Mg, K, Na, Al, B, Ba, Cd, Cu, Cr, S, Se, Sn, Fe, Mn, Mo, Ni, P, Zn	Hemp varieties	1.0 g of lyophilized sample, ground, digested in microwave system with 10 mL of 69% HNO_3_. The digestion program was from 20 °C to 140 °C for 30 min, then kept for 50 min at 140 °C	[57]
Ca, Cu, Fe, Mn, Mg, Zn	Sprouts	0.5 g of freeze-dried sprouts, ground, digested in microwave system with 10 mL of concentrated HNO_3_. After cooling down, 6 M HCl were added	[58]
As, Pb, Hg, Ni, Cd, Cu, Cr, Zn	Corn and soybean	1 g of grounded sample digested with 15 mL of a mixture of HNO_3_ 65%, H_2_SO_4_ 98%, and HCl 36% (5:1:1 *v*/*v*) heated at 80 °C until obtaining a clear solution	[59]
Al, Ba, Ca, Cu, K, Fe, Li, P, Mg, Mn, Na, Sb, Se, Zn	Vegetable oils	0.5 g of sample digested with 3 mL of HNO_3_ 65% and 3 mL of H_2_O_2_ 30% in microwave-assisted conditions	[60]
Al, Ba, Cu, Ca, K, Fe, Na, Ni, Mg, Mn, S, P, Sr, Zn	Chocolate and cocoa	1 g of sample mixed with 9 mL of HNO_3_ 65% and then heated in a water bath at 95 °C for 1 h, transferred, and diluted to 25 mL with deionized water	[61]
Cd, Cr, Cu, Co, Mn, Ni, Zn, Pb	Sauces from different ingredients	10 mL of sample mixed with 10–15 mL aqua regia, kept 1 h. Then, it was added to 100 mL water and heated on the hot plate at 150 °C.	[62]
Al, Ag, Ba, B, Bi, Ca, Co, Cd, Cu, Cr, Fe, Mn, Mg, Pb, Ni, Tl, Zn	Spices	0.2 mg of sample mixed with 6 mL of HNO_3_ 65% and 1 mL of H_2_O_2_ 30% and heated for 90 min at 120 °C in a heating block. After cooling down at room temperature, the digested sample was diluted 25 mL	[63]
Pb, As, Cd, Cu, Zn	Fruit juices (apple, grape, peach, orange, mango, pineapple)	2 mL of sample added to 20 mL mixture of HNO_3_ 65% and H_2_O_2_ 30% at a ratio of 9:1, *v*/*v*, stirred 10 min at room temperature, and then heated at 180 °C for 15 min. Samples were digested in microwave oven at 1800 W for 27 min	[64]
As, Ca, Cu, Cr, Cd, P, K, Fe, Mg, Mn, Pb, Ni, Zn	Legumes (*Phaseolus* spp., *Vicia* spp., *Pisum* spp. and *Lathyrus* spp.)	0.5 g of flour sample mixed with 5 mL of HNO_3_ 65% and 2 mL of H_2_O_2_ 30%. A three-step microwave digestion program with a total time of 40 min at 800 W was applied	[65]
As, Cd, Cr, Cu, Pb, Fe, Mn, Ni, Zn,	Vegetables	0.2 g of sample mixed with 4 mL of conc. HNO_3_, heated in a water bath for 150 min up to 100 °C. Then the sample was cooled at room temperature and 0.2 mL of H_2_O_2_ 30%, and let to react 30 min	[66]

**Table 2:** Examples of combustion and wet acid digestion of ashes for element extraction from plant samples.

Analytes	Type of Samples	Digestion Method	References
Ca, Cu, K, Mg, Mn, Na, P, Zn Fe	*Gynandropsis gynandra*	0.5 g of powdered sample was burned for 2 h in a furnace at 550 °C. The ashes were digested with 10 mL HNO_3_:HCl, 1:3 ratio mixture on a hot plate	[28]
Ba, Be, Bi, Ca, Co, Cs, Cu, Mg, Mn, Na, K, P, Pb, Ni, Rb, Sr, Mo, Th, U, Zn, REEs	Botanical samples	0.2 g sample incinerated using infrared assisted heating in quartz tubes. 10 mL of 10% HNO_3_ was added to dissolve the ash	[55]
P, K, Na, Ca, Mg, Fe	Beetroot (*Beta vulgaris* L.)	Sample was burned in an oven at 550 °C for 24 h. The incineration residue was then extracted with HCl (50%, *v*/*v*) and HNO_3_ (50%, *v*/*v*)	[85]
Al, Co, Cd, Cu, Cr, Fe, Mo, Mn, V, Pb, Zn	*Medicago sativa* L., *Cynodon dactylon* L., *Corchorus olitorius* L., *Avena sativa* L., *Cynara scholymus* L.	2.0 g of powdered sample was burned for 3 h in a furnace at 550 °C. 60 mL aqua-regia was added and heated on hot plate at 100 °C	[86]
As	Black radish, lettuce, black salsify, savoy cabbage, parsnip, swede turnip	1 g of powdered sample decomposed in an oxidizing gas mixture at 400 °C. 20 mL 1.5% HNO_3_ was added to dissolve the ash	[87]
Al, As, Zn, B, Cd, Cu, Co, Pb, Fe, Se	Baby food	10 g of sample mixed with 5 mL of 65% HNO_3_, heated on hot plate until acid evaporation, then burned at 450 °C 24 h. The ash dissolved in 1.5% HNO_3_	[88]

**Table 3:** Examples of procedures for element extraction with complexing reagents and green solvents.

Analytes	Type of Samples	Digestion Method	References
Al, Ca, As, Co, Cd, Cr, Fe, Cu, K, Mg, Na, Mn, P, Si, Pb, Zn	10 medicinal plant species	0.5 g of plant dried sample extracted with 20 mL ethanol/water solution (50% (*v*/*v*))	[25]
Ag, Al, Ba, B, Ca, Co, Cu, Cr, Fe, Mg, Mo, Mn, Ni, Na, Pb, Ti, Sn, V, K, Zn	Oil samples	5 g oil mixed with 0.5 g of DES (choline chloride and hydrogen donors: tartaric, citric, benzoic, oxalic, acetic, malonic, malic, formic, maleic, succinic, adipic, boric, lactic, ascorbic, gallic, and mandelic acids; 1;4-butanediol; glycerol; sorbitol; ethylene glycol; triethylene glycol; benzamide; urea; thiourea; fructose; glucose; sucrose; maltose	[92]
Ca, Cu, Ba, Na, K, Fe, Mn, Mg, Mo, Pb, Ni, Sn, V, Zn,	Tobacco, lettuce	100 mg of plant sample mixed with 0.5 g of DES (choline chloride, and malic acid, 1:1) at 70 °C	[94]
Al, Ag, Ba, Cd, Cu, Cr, Fe, Li, K, Ni, Mg, Pb, Mn	Oil samples	DES (ethylene glycol and choline chloride, ratio (2:1)) and aerosol phase extraction method	[95]
As, Cd, Ca, Cu, K, Fe, Na, Mg, Mn, P, Zn	Vegetables	90 mg sample mixed with 9 mL natural deep eutectic solvents (xylitol, citric acid, malic acid) in ultrasound-assisted conditions	[96]
Al, Cr	Vegetables	Ionic liquid dispersive liquid–liquid microextraction, based on anionic chelate complexes formation between Al(III) and Cr(VI) with o-hydroxy azo dye, and extraction of the ternary complexes	[97]
Cd	Oil samples	Dispersive solid phase extraction with stearic acid coated with Fe_3_O_4_ nanoparticle as adsorbent	[98]
As, Cd, Cu, Fe, Pb, Mn, Ni, Zn	Oil samples	0.1 g oil mixed with 10 mL of diluted acids mixture 1% HNO_3_/0.2% HCl. Shaken by vortex, then ultrasound extraction	[99]
As, Cd, Co, Cr, Sb, Tl, Pb	Vegetables	Ultrasound-assisted cloud point extraction (UA-CPE) combined with dispersive μ-solid phase extraction (D-μ-SPE) for preconcentration of metals. A nanocomposite compound Mg/Al-LDH@CNTs was synthetized and used as solid phase	[100]
As, Cd, Hg, Pb	Drug samples	A combination of dispersive liquid–liquid microextraction using deep eutectic solvent (NADES) as extractant combined chemical vapor generation	[101]
Se	Cereal and biofortified samples	DES (choline chloride (ChCl) as hydrogen bond acceptor, and phenol (PhOH) as hydrogen bond donator) at different mole ratios of ChCl: PhOH = 1:1, 1:2, 1:3 and 1:4	[102]

**Table 4:** Examples of digestion methods used in bioaccesibility studies on plant samples.

Analytes	Type of Samples	Digestion Method	References
P, K, Na, Ca, Mg, Fe	Beetroot (*Beta vulgaris* L.)	Sample digestibility assessed using the in vitro digestion method: oral (pH 7), gastric (pH 3), intestinal (pH 7), and digested (D) phases.	[85]
Cu, Mn, Fe, Ni, Zn	Hazelnut	Sample digestibility assessed using the in vitro digestion method: stomach (pH 2.5), Intestine 1 (pH 7), Intestine 2 (pH 7)	[105]

**Table 5:** Figures of merit reported in plant analysis by ICP-OES.

Analytes	Type of Samples	Analytical Performances	References
Hg, Pb	Vegetables, herbs	Vegetable and/or herb samples spiked at concentrations of 15, 25, 75, 150, 250, 500, and 750 μg/mL for recovery test	[26]
As, Co, Ca, Cu, Cr, Fe, Mn, Mg, Pb, Ni, Se, Zn	*Laportea alatipes*	CRM White clover (BCR 402) analyzed for quality assurance	[27]
As, Cd, Cr, Cu, Ni, Mn, Fe, Pb, Zn	Sweet potato	CRM INCT-CF-3–corn flour, analyzed for quality assurance	[33]
As, Cd, Co, Cr, Cu, Mn, Mo, Ni, Pb, Sb, and Sn	Tomato, onion, pepper, spinach, carrots, lettuce, marrow squash	CRM NCS ZC85006 tomato analyzed. Student’s t-test at the 95% level, indicated the results consistent with the certified values (recoveries between 93–103%)	[38]
Cd, Pb, As, Hg	Okra, tomato, pumpkin, potato, cabbage, eggplant, spinach	Fortified potato samples analyzed. Obtained recoveries varied in the range 83–103%, RSD, varied between 7–14%	[39]
Ca, K, Mg, P, Na, B, Fe, Mn, Zn, Al, Sr, Co, Cu, Ni, Se, V, As, Cd, Cr, Pb, Sb, Sc, Y	Kale, rapeseed	CRM apple (Malus domestica Borkh) leaves NIST-SRM 1515 was analyzed. Recoveries varied from 84–118% of certified values	[40]
As, Cd, Pb, Cr	Ten species of edible plant samples	CRM analyzed, recoveries in the recoveries were in the range of 96–100%	[45]
As, Pb, Hg, Ni, Cd, Cu, Cr, Zn	Corn, soybean	LODs 0.001–0.005 mg/kg; LOQs 0.003–0.015 mg/kg Inter-day precision between 3.2–6.4%	[59]
Al, Ba, Ca, Cu, K, Fe, Li, P, Mg, Mn, Na, Sb, Se, Zn	CRM tomato leaves	Recoveries in the range of 75–101.5%.	[60]
Al, Ba, Cu, Ca, K, Fe, Na, Ni, Mg, Mn, S, P, Sr, Zn	Cacao	Use of spiked solutions for percentage recovery	[61]
Cd, Cr, Cu, Co, Mn, Ni, Zn, Pb	SRM baking chocolate	Recoveries ranged from 98.6–101.2%	[62]
Al, Ag, Ba, B, Bi, Ca, Co, Cd, Cu, Cr, Fe, Mn, Mg, Pb, Ni, Tl, Zn	Spices	Two emission lines were verified for each element for selectivity evaluation. LOQs were in the range of 0.27 to 19.83 mg/kg. Recoveries were between 82.0 and 117.5%	[63]
Pb, As, Cd, Cu, Zn	Fruit juices (apple, grape, peach, orange, mango, pineapple)	LODs between 0.03 and 0.92 µg/L Recoveries between 93 and 99%	[64]
As, Ca, Cu, Cr, Cd, P, K, Fe, Mg, Mn, Pb, Ni, Zn	Legumes	LODs in the range 0.027 mg/L (Cd)–0.076 mg/L (P) Recovery 88–106%	[65]
As, Cd, Cr, Cu, Pb, Fe, Mn, Ni, Zn	Vegetables	LODs in the range 0.049 ppm (Cd)–0.564 mg/L (Cr)	[66]
Al, As, Zn, B, Cd, Cu, Co, Pb, Fe, Se	CRMs with plants and food matrices	LODs 0.001–3.655 mg/L; recoveries in the range of 93.1–102.7%	[88]
Cd, Ni, Pb, Hg	Food samples	Recoveries ranged from 95.0–106.0% LOQ between 2.1 and 14.8 µg/kg	[89]
Al, Ba, B, Cd, Co, Cu, Cr, Fe, Pb, Mn, Li, Mo, Ni, Sr, V, Zn	Vegetable sausages	LOQs between 0.001 mg/L (Cd, Pb) and 0.013 mg/L (Li)	[90]
As, Cd, Cu, Fe, Pb, Mn, Ni, Zn	Oil	LODs 0.002–0.036 mg/L	[99]
As, Cd, Co, Cr, Sb, Tl, Pb	Vegetables	LODs in the range of 90–150 ng/L by preconcentration using micro-solid phase extraction. Recoveries between 97 and 99.3%	[100]

## Data Availability

Not applicable.
